# Antimicrobial Pressure of Ciprofloxacin and Gentamicin on Biofilm Development by an Endoscope-Isolated *Pseudomonas aeruginosa*

**DOI:** 10.5402/2013/178646

**Published:** 2012-08-28

**Authors:** Idalina Machado, Joana Graça, Hélder Lopes, Susana Lopes, Maria O. Pereira

**Affiliations:** Centre of Biological Engineering, Institute for Biotechnology and Bioengineering (IBB), University of Minho, Campus de Gualtar, 4710–057 Braga, Portugal

## Abstract

This work aims at characterizing endoscope biofilm-isolated (PAI) and reference strain *P. aeruginosa* (PA) adhesion, biofilm formation and sensitivity to antibiotics. The recovery ability of the biofilm-growing bacteria subjected to intermittent antibiotic pressure (ciprofloxacin (CIP) and gentamicin (GM)), as well as the development of resistance towards antibiotics and benzalkonium chloride (BC), were also determined. The capacity of both strains to develop biofilms was greatly impaired in the presence of CIP and GM. Sanitization was not complete allowing biofilm recovery after the intermittent cycles of antibiotic pressure. The environmental pressure exerted by CIP and GM did not develop *P. aeruginosa* resistance to antibiotics nor cross-resistance towards BC. However, data highlighted that none of the antimicrobials led to complete biofilm eradication, allowing the recovery of the remaining adhered population possibly due to the selection of persister cells. This feature may lead to biofilm recalcitrance, reinforcement of bacterial attachment, and recolonization of other sites.

## 1. Introduction

*Pseudomonas aeruginosa* is an opportunistic pathogenic bacterium [1] widely investigated for its high incidence and extraordinary ability to form strong biofilms in clinical equipment, medical devices, and wounds [2, 3]. This microorganism is commonly associated with nosocomial infections and is a leading cause of severe and life-threatening infections, especially in immunosuppressed hosts [4]. 

*P. aeruginosa* is one of the most common microorganisms transferred by bronchoscopes, being the most frequent in gastrointestinal endoscopy [5]. Flexible endoscopes undergo repeated rounds of patient use and reprocessing. Studies related to endoscope contamination have reported the presence of biofilms on the inner surface of endoscope channels [6, 7], highlighting the importance of effective measures for cleaning and disinfection in endoscope reprocessing. Biofilm removal is a crucial step to prevent lapses in reprocessing, being thus of clinical relevance in endoscopy [5, 8]. Biofilms represent a reservoir of pathogenic bacteria that can detach, resume their planktonic state, and contaminate new surfaces and patients. Moreover, microbial biofilms are notorious for their high level of resistance towards antibiotic and biocide treatments [9]. Bacteria within biofilms can easily live in the presence of high antibiotic concentrations similar to the ones that are prescribed during the course of therapies [10, 11]. Biofilm resistance mechanisms involve not only the reaction-diffusion limitation of antimicrobial access to the biofilm-entrapped bacteria [12, 13] but also the expression of spatially heterogeneous, less susceptible phenotypes, caused either by growth as a biofilm per se [14] or through the expression of high cell density [15] or starvation phenotypes [16]. Antibiotics and biocides are frequently used in hospitals with the purpose of controling the growth of, or to kill bacteria in, respectively, infection control and sanitation. The use of certain active substances in biocides in various settings may contribute to the increased occurrence of antibiotic-resistant bacteria. Cross-resistance between biocides and antibiotics and between different antibiotics has been reported previously [17], and there are several studies suggesting that if two antimicrobial compounds have similar mechanisms of action, they may also share resistance mechanisms [18]. It has also been demonstrated that highly antibiotic-resistant clinical isolates of Gram-negative bacteria are generally more resistant to disinfectants [19]. Although there is much concern regarding the risks of antibiotic resistance induced by the use of and resistance to biocides, there is a lack of studies evaluating the performance of disinfectants after bacterial exposure to antibiotics. Studies related with the use of sessile bacteria to assess the efficacy of antibiotics and biocides are even lesser, even though biofilm formation is an important aspect of many bacterial diseases. These biofilm tests should also include bacteria isolated from real scenarios as they can present genetic diversity and thus possess distinguishing virulence factors. The pathogenesis of *P. aeruginosa* is attributed to the production of several cell-associated and extracellular virulence factors that arise under certain environmental conditions [20].

In the present work, the phenotype (early-stage adhesion, biofilm formation, and sensitivity to antimicrobials) of *P. aeruginosa* isolated from a biofilm formed on an endoscope was determined and compared with the reference strain. Furthermore, this study was also undertaken to determine whether exposure of *P. aeruginosa* biofilms to intermittent cycles of antibiotic chemotherapy (ciprofloxacin (CIP), and gentamicin (GM)) could lead to regrowth and potential resistance and cross-resistance towards a disinfectant, BC, CIP and GM. 

## 2. Methods

### 2.1. Test Organisms and Culture Conditions

*P. aeruginosa *(ATCC 10145) (PA) and *P. aeruginosa* isolated (PAI) from a biofilm formed in a medical device (gastrointestinal endoscope) were preserved at −80 ± 2°C in 10% glycerol stocks. Prior to each experiment, bacterial cells were grown on Tryptic Soy Agar (TSA, Merck) plates for 24 h, at 37°C.

To prepare the bacterial suspensions, one colony of each strain of *P. aeruginosa* (PA or PAI) was collected from the TSA plates and grown in Tryptic Soy Broth (TSB) for 24 h at 37°C, with agitation. Subsequently, bacteria were harvested by centrifugation and washed twice with sterilized ultrapure water (UP). Standardized cell suspensions were prepared in TSB unless otherwise stated.

### 2.2. Antibacterial and Antibiotic Agents

Ciprofloxacin (CIP), a broad-spectrum synthetic chemotherapeutic antibiotic of the fluoroquinolone drug class, used clinically to treat *P. aeruginosa *infections, was purchased from Fluka. Gentamicin (GM), an aminoglycoside antibiotic, used to treat many types of bacterial infections, particularly those caused by Gram-negative organisms, was purchased from Sigma. Benzalkonium chloride (BC), a quaternary ammonium compound, widely used in clinical disinfectant formulations, was obtained from Calbiochem.

The antibiotics concentrations used in the present work were determined using as reference the 3x MBC [21] of each product for the reference strain. So, for biofilm disturbance cycles and biofilm treatment, a concentration of 3 mg/L of CIP and 10 mg/L of GM were used. Concerning BC, the MIC concentration of 360 mg/L was used.

### 2.3. Early Bacterial Adhesion

In order to determine the adhesion ability of both strains, a parallel plate flow chamber (PPFC) and image analysis system as described by Sjollema et al. [22] were used to study the early-stage bacterial adhesion and detachment. Briefly, the PPFC consists of a nickel-coated frame measuring 16 × 8 × 1.8 cm. Teflon spacers were placed between the plates, to separate them by 0.06 cm.

The PFFC device was mounted in a phase contrast inverted microscope (Diaphot 300; Nikon) equipped with a 40x ultralong working distance objective. The images were acquired in a CCD camera (AVC, D5CE; Sony) connected to the microscope and coupled to an image analyser (Image Proplus 4.5; Media Cybernetics).

Prior to each experiment, all tubes and the flow chamber were filled with sterile phosphate buffered saline (PBS), taking care to remove air bubbles from the system. To assess the rate of attachment of reference strain and isolated *P. aeruginosa* on polystyrene (PS), bacterial suspensions were put to circulate through the PPFC at 0.020 mL/s for 30 min to allow surface colonization under flow. Then, PBS was circulated through the equipment, at the same rate, to remove unattached and weakly adhering cells and thus to evaluate the detachment of the bacterial cells.

The initial increase in the number of adhering microorganisms with time was expressed as the so-called initial deposition rate [*j*_0_, cells/(cm^2^ s)], that is, the number of adhering microorganisms per unit area and time. The number of adhering microorganisms after 30 min of bacterial suspension flow [*n*_30 min _, cells/(cm s)] and the number of microorganisms after PBS passage [*n*_60 min _, cells/(cm s)] were also determined. The rate of detachment (%) denotes the percentage of *P. aeruginosa* cells that were detached upon the passage of PBS trough the flow chamber. 

### 2.4. Biofilm Formation

The biofilm formation ability of PA and PAI on polystyrene (PS) was inspected along time (24, 48, 72, and 96 h) using the microtiter plate test developed by Stepanović et al. [23]. Briefly, cell suspensions of both strains were diluted to obtain a final concentration of 1.0 × 10^7^ cfu/mL. Afterwards, 200 *μ*L/well of the bacterial suspension was transferred to sterile 96-well flat-bottom tissue culture plates (Orange Scientific). All the plates were incubated at 37°C, during 24 h for biofilm development, with agitation.

After 24 h of biofilm growth, the supernatant containing planktonic cells and media was removed. The wells were re-filed with fresh TSB, and this process of supernatant removal and media filling was repeated for every 24 h until 96 h of biofilm formation. 

After 24, 48, 72, and 96 h of growth, biofilms were characterized in terms of biomass, respiratory activity, and number of viable biofilm-entrapped cells.

### 2.5. Biofilm Disturbance and Recovery

To assess whether the presence of antibiotics could interfere with the establishment of biofilms by both strains on PS surfaces, biofilms were allowed to form for 24 h in the presence of both antibiotics. To ascertain the postantibiotic effects (PAEs) of CIP and GM on *P. aeruginosa*, those challenged biofilms were later subjected to intermittent cycles of antibiotic chemotherapy with CIP and GM. 

Biofilms were formed in microtiter plates with cell suspensions of both strains at a final concentration of 1.0 × 10^7^ cfu/mL prepared in TSB containing CIP or GM in a final concentration of, respectively, 3.0 mg/L or 10 mg/L. Each cycle of antibiotic treatment was followed by a recovery period of 24 h, where biofilms only developed in 200 *μ*L of fresh TSB. 

After 24, 48, 72, and 96 h the content of each well was removed and biofilms were washed and phenotypically characterized.

### 2.6. Biofilm Susceptibility

The 96-hour-old biofilms untreated and submitted to the intermittent antibiotic chemotherapy with CIP and GM were inspected regarding their susceptibility towards the same antibiotics as well as the potential occurrence of cross-resistance towards the disinfectant BC.

In order to determine the biofilm response after antibiotic therapy, biofilms were treated with 200 *μ*L per well of 360 mg/L of BC, 3 mg/L of CIP or 10 mg/L of GM for 30 min. Nontreated wells were filled with 200 *μ*L of UP sterilized water. After that, the content of each well was removed and biofilms were washed with 200 *μ*L with ultrapure sterilized water (UP) being reserved for posterior analysis.

### 2.7. Biofilm Analyses

#### 2.7.1. Biofilm Mass and Activity

Biomass of *P. aeruginosa* biofilms was quantified by crystal violet (CV) staining method adapted from Stepanović et al. [23]. For that, the plates containing the biofilms were left to air-dry for 30 min, and 200 *μ*L of 98% methanol was transferred to each well in order to fix the remaining attached biofilm, for 15 min. Afterwards, the plates were emptied and left to air dry. The fixed bacteria were stained with 200 *μ*L of 1% (w/v) CV (Gram's staining; Merck) per well, for 5 min. After this staining step, plates were washed with running tap water, air dried, and filled with 200 *μ*L of 33% (v/v) of acetic acid (Merck) in order to resolubilize the CV bound to the adherent bacteria. The quantitative analysis of biofilm production was performed through the measurement of optical density at 570 nm (OD_570_), of each well using a microtiter plate reader (Bio-Tek Synergy HT, Isaza), the biofilm mass being presented as OD_570_. Control experiments to avoid false results were also performed. When the optical density was higher than 1.0, samples were diluted with 33% (v/v) of acetic acid. For each condition tested, eight different wells were used to quantify the mass of biofilms.

Biofilm activity was determined with 2,3-bis(2-methoxy-4-nitro-5-sulfo-phenyl)-2H-tetrazolium-5-carboxanilide (XTT) colorimetric method as described by Stevens and Olsen [24], with some modifications. After biofilm development and treatment, 200 *μ*L of a combined solution of XTT (Sigma) and PMS (phenazine methosulfate) (Sigma) was added to each well in order to obtain a final concentration of 150 mg/L of XTT and 10 mg/L of PMS. After that, plates were incubated at 37°C for 3 h, with agitation, in the dark. Biofilm activity was determined through measurement of the optical density at 490 nm of the liquid content of each well using a microtiter plate reader, biofilm activity being presented as OD_490_. Control tests, were also carried out, in order to avoid misleading results. For each condition tested, 8 different wells were used to determine biofilm activity. 

#### 2.7.2. Biofilm Cell Enumeration

In order to determine the number of biofilm-entrapped viable bacteria, biofilm suspensions was prepared. Two-hundred microliters of UP sterilized water being were added to each well, the wells-attached biofilms removed by ultrasonic bath in a Sonicor SC-52 (Sonicor Instruments) operating at 50 kHz, during 6 min. Afterwards, the bacterial suspensions of each 5 wells per condition were collected and gently vortexed for two min to disrupt possible cell aggregates (these parameters were previously optimized in order to promote the complete removal of all the biofilm-attached cells without lysis). Bacterial suspensions were serially diluted, plated on TSA, and incubated at 37°C in an aerobic incubator for 24 h. The number of colony forming units (cfu) was enumerated, being the biofilm cell numbers presented as log_10_ (cfu/cm^2^).

### 2.8. Statistical Analysis

Statistical analysis was performed using GraphPad Prism, version 4.0 software for Macintosh. Normality of data distribution was tested by the Kolmogorov-Smirnov method. Statistical significance values of the groups' means of biofilm mass, biofilm activity, and cell number were evaluated using a one-way analysis of variance. Subsequent comparisons were performed using Tukey's post hoc test. Two-way analysis of variance with Bonferroni post hoc test were used to compare means of biofilms obtained after 96 h, and after CIP and GM regrowth cycles after treatment. The statistical analyses performed were considered significant when *P* < 0.05.

## 3. Results

### 3.1. Attachment and Detachment Monitoring

The results obtained with early bacterial adhesion assay (Table 1) revealed that both strains had high ability to adhere to surfaces, but the rate of cell deposition (*j*_0_) of the isolated strain was higher than that of PA (*P* < 0.05). After 30 min of adhesion, the number of adhered cells (*n*_30 min _) is about twice for the isolated strain (*P* < 0.05). Also, the number of PAI cells that remained attached in the PS surface (*n*_60 min_) was the double of PA cells (*P* < 0.05), the percentage of detachment of PA cells after PBS passage being of about 20%.

### 3.2. Biofilm Formation

**Figure 1:**
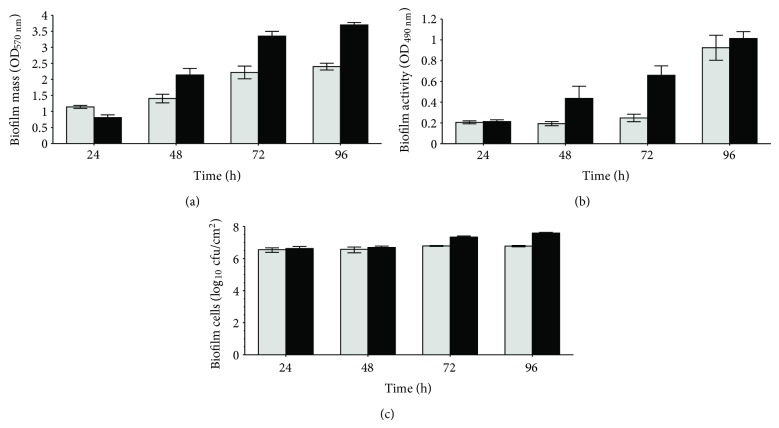
Biomass (OD_570 nm_) (a), metabolic activity (OD_490 nm_) (b) and number of cultivable cells (c) of *P. aeruginosa* ATCC (light grey) and *P. aeruginosa* isolated strain (black) biofilms. Biofilms where grown in TSB and characterized at 24, 48, 72 and 96 h. Bars represent the average of 3 independent repeats ± SD.

In order to examine the biofilm formation ability of both PAI and PA strains, the biofilm phenotype was characterized in terms of mass, activity and number of cells after 24, 48, 72, and 96 h of growth (Figure 1). In general, data showed that mass and activity of biofilms increased along time, whereas the number of biofilm-entrapped cells was approximately in the same magnitude, for all the time periods of biofilm formation. Comparing both strains, Figure 1 shows that, in general, PAI gave rise to biofilms with more mass than PA (*P* < 0.05) and activity (*P* < 0.05). The number of biofilm cells (Figure 1(c)) was identical to those quantified for PA biofilms, except for 72-hour-old and 96-h-old biofilms in which there was an increase in the number of cells (*P* < 0.001). These results indicate that both strains are good biofilm producers although the isolate stands out relatively to the collection.

### 3.3. Biofilm Disturbance and Recovery

**Figure 2:**
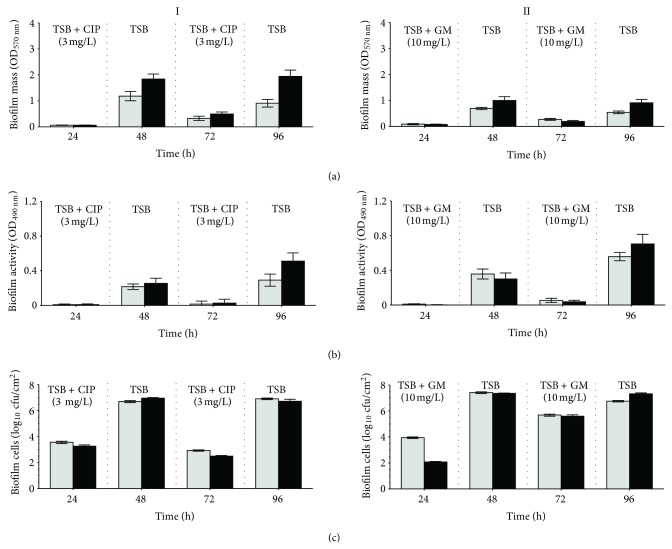
Biomass (OD_570 nm_) (a), metabolic activity (OD_490 nm_) (b), and number of cells (c) of *P. aeruginosa* ATCC (light grey) and *P. aeruginosa* isolated strain (black) biofilms. Biofilms were continuously grown in TSB for 96 h with the application of intermittent cycles for 24 h of 3 mg/mL of CIP (I) and 10 mg/mL GM (II).

The presence of CIP and GM in the first 24 h of biofilm development clearly hampered the establishment of biofilms by both strains on PS surfaces (Figure 2). In fact, the phenotype of PA and PAI biofilms grown for 24 h under antibiotic pressure was characterized by a large decrease in biofilm mass and activity (about 95%) and a reduction of about 4 log in the number of viable biofilm-entrapped cells. However, it must be emphasized that a considerable number of cells remained viable on the surfaces. These data revealed that CIP and GM have a significant in vitro anti biofilm formation activity, this effect being similar for both strains.

After a recovery period of 24 h, where growth occurred in absence of antibiotics, those remaining less dense biofilms recovered its levels of biomass, activity and number of cells (Figure 2). Biofilm recovery after CIP pressure (Figure 2I) gave rise to PAI biofilms with higher values of biomass (*P* < 0.001), activity, and viable cells (*P* < 0.01), when compared with PA biofilms. This trend was similar to that observed in the 48-hour-old biofilms formed without any stress factor (Figure 1). Regarding the post-GM effect (Figure 2II), the superiority of the biofilms formed by the isolated strain is no longer evident as PAI biofilms only showed higher biomass (*P* < 0.001).

The second cycle of biofilm growth under antibiotics pressure clearly reduced the mass, activity, and number of biofilm-encased cells, for both strains (Figure 2). However, these reductions were lower than those obtained after the first cycle of antibiotic treatment, mainly when GM was used. These results showed that both antibiotics have ability to disturb established *P. aeruginosa* biofilms causing its removal and inactivation. Nevertheless, as Figure 2 shows, this sanitation was not total, allowing biofilm regrowth during the second recovery period. In fact, the resulting 96-hour-old biofilms recuperated again its levels of biomass and activity, although they are far from those observed in biofilms developed by both strains in the absence of antibiotic stress (Figure 1). The numbers of viable biofilm-cells were also restored reaching, however, values in the same order of magnitude of those determined in the 96-hour-old biofilms formed in TSB. Comparing the behaviour of both strains, in general, PAI biofilms showed higher biomass, activity and number of cells than the biofilms formed by the reference strain.

The postantibiotic effects observed after the second cycle of antimicrobial treatment is similar to that observed after the first biofilm growth under antibiotic pressure, except for biofilm activity (Figure 2). In fact, the activity of the 96-hour-old biofilms was higher than those observed after the first 24-h recovery period, specially for biofilms grown under GM pressure (*P* < 0.001) and for those developed by PAI (*P* < 0.001) (Figure 2(b)).

The overall results highlighted that both antibiotics have good anti-biofilm characteristics and ability to remove and inactivate established *P. aeruginosa* biofilms. Nevertheless, sanitization was not complete allowing the resumption of the biofilms immediately following antibiotic pressure.

### 3.4. Biofilm Susceptibility

**Figure 3:**
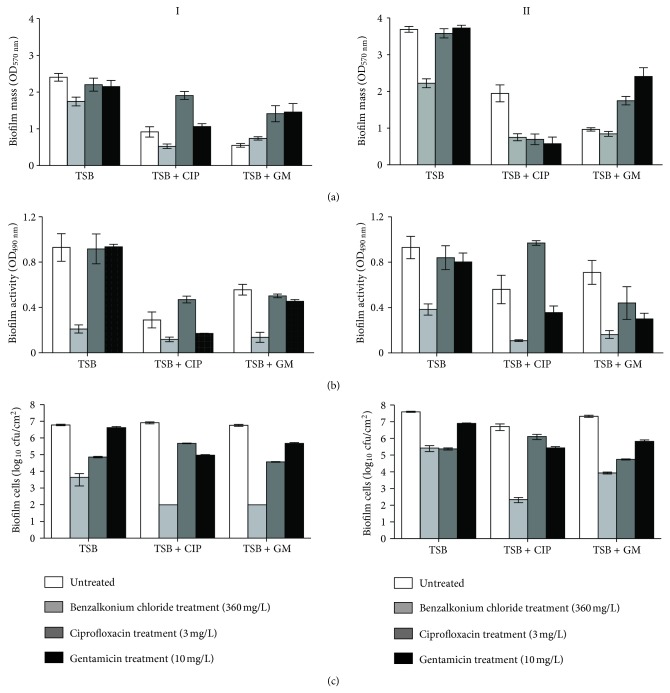
Biomass (OD_570 nm_) (a), metabolic activity (OD_490 nm_) (b), and number of cultivable cells (c) of 96-hour-old biofilms formed by *P. aeruginosa* ATCC (I) and *P. aeruginosa* isolated strain (II). Biofilms were continuously grown in TSB for 96 h (TSB) or with the application of intermittent cycles for 24 h of 3 mg/mL of CIP (TSB + CIP) and 10 mg/mL GM (TSB + GM). Normal biofilms (control, white) and treated with BC (light grey), CIP (dark grey), and GM (black).

The susceptibility of the 96-h-old biofilms, subjected to the intermittent cycles of antibiotic pressure, towards antibiotics (GM and CIP) and biocide (BC) treatment can be observed in Figure 3. In the range of conditions tested, the 96-h-old biofilms formed by both strains in TSB were practically tolerant to the action of antibiotics and susceptible to the toxic effect of BC. In fact, only treatment with BC of PA and PAI biofilms promoted a significant reduction of biomass (Figure 3(a)) (*P* < 0.001), respiratory activity (Figure 3(b)) (*P* < 0.001), and number of viable cells (Figure 3(c)) (*P* < 0.001).

The 96-hour-old biofilms formed by both the ATCC and the isolated strain under intermittent cycles of CIP or GM pressures were also clearly disturbed by the action of BC. It appears that antibiotic pressure during biofilm growth gave rise to biofilms more susceptible to the antimicrobial action of benzalkonium chloride.

The response of PA biofilms towards CIP and GM treatment depended on the antibiotic used. The PA biofilms challenged by CIP pressure (Figure 3I) are practically indifferent to the posterior aggression with the same antibiotic, as the values of biofilm mass (Figure 3(a)I) and activity (Figure 3(b)I) were higher than those observed in biofilms without treatment (*P* < 0.01), although there was a reduction in the number of viable biofilm-cells (*P* < 0.001) (Figure 3(c)I). The response of these biofilms to the action of GM was somewhat different, as GM caused the reduction of biofilm metabolic activity and number of biofilm-entrapped cells.

The 96-h-old biofilms formed under GM intermittent pressure appeared to be more tolerant towards both antibiotics as the aggression of CIP and GM for 30 min only decreased the number of viable biofilm cells (*P* < 0.001) (Figure 3(c)I).

Regarding PAI biofilms (Figure 3II), those developed under CIP pressure were more sensitive to the action of GM than CIP since only significant reductions in biofilm mass (*P* < 0.001) (Figure 3(a)II) respiratory activity (*P* < 0.001) (Figure 3(b)II), and number of viable cells (*P* < 0.001) (Figure 3(c)II), were observed after GM treatment. The actions of CIP and GM against the 96-hour-old PAI biofilms, previously grown under GM pressure, were quite similar as both antibiotics increased the biomass accumulated on the PS surfaces (Figure 3(a)II) (*P* < 0.05) and slight decreased the biofilm activity (Figure 3(b) II) (*P* < 0.05) and the number of the biofilm cells (Figure 3(c)II)  (*P* < 0.001).

Based on Figure 3, it can be stated that none of the conditions led to complete sanitation of the biofilms, with, in general, the action of the antimicrobials being more effective in bacteria inactivation than in biofilm removal. Data also highlighted that cross-resistance between antibiotics and the biocide did not occur.

## 4. Discussion

Adhesion and biofilm formation are two important aspects of many bacterial diseases, especially those related with medical devices [25], as flexible endoscopes. When biofilms are identified as the main cause of infection, treatment becomes very difficult since bacteria within biofilms adopt special features that confer them increased resistance to antimicrobial agents [9]. This resistance usually makes sessile microorganism more difficult to kill and remove from surfaces than planktonic counterparts. Furthermore, in many cases incomplete removal of the biofilm allows it to quickly return to its equilibrium state.

In this work, some phenotypic characteristics (early-stage adhesion, biofilm formation ability and sensitivity to different antimicrobials) of *P. aeruginosa* isolated from an endoscope were inspected.

The ability to adhere of the isolated strain was superior to that of reference strain as  *j*_0_  and  *n*_30 min _  are around two-fold higher than those observed with the reference strain. Besides revealing higher rate of adhesion (*j*_0_), the isolated strain also showed higher number of cells adhered to the surface after 30 min of contact (Table 1). Furthermore, the strength of adhesion of this bacterium on PS surfaces was stronger as PBS circulation failed on cell detachment. In fact, for the reference strain the detachment of cells was around 20%, while for the isolated strain no significant cell detachment was observed. Knowing that PAI was obtained from a real biofilm formed on an endoscope, it is conceivable to speculate that the isolated strain has been exposed to mechanical and chemical stress conditions, namely, during endoscope reprocessing. So, as a survival strategy, this isolated strain may have acquired phenotypic and physicochemical changes that allowed it to adhere easily on PS and with superior strength. In fact, other authors [26] reported that organisms isolated from any given niche, medical, environmental, or industrial, have different mechanisms of adhesion and retention, mainly due to changes in their structural components, such as pili, fimbriae, and adhesive surface proteins that have adapted differently over time through selective pressures. Furthermore, exposure to antimicrobials may as well induce changes in cell surface hydrophobicity and surface charge that can alter bacterial adhesion properties [27].

The prominence of PAI was also visible in terms of biofilm formation ability as it has developed biofilms with more biomass, respiratory activity and number of cells than PA (Figure 1). This feature together with the greatest capacity to adhere allows to speculate that this isolated strain is more pathogenic than ATCC strain, as bacterial attachment and biofilm formation are considered important virulence factors of bacterial pathogens [20, 28]. In fact, the formation of thick biofilms gives bacteria, amongst other advantages, protection from external aggressions, as host defences and antimicrobials, due to the lipopolysaccharides that constitute the EPS matrix [29]. This EPS matrix acts also as a diffusion barrier that can reduce antimicrobial efficacy by diminishing its penetration into the deeper layers of the biofilm. Protected within this niche, bacteria can detach, proliferate and furthermore disseminate in large amounts making possible the spread of pathogens [30].

Although the isolated strain develops thicker biofilms, it must be referred that luckily the capacity of both strains to develop biofilms was greatly impaired in the presence of antibiotics. The concentration of antibiotics used to cause antimicrobial stress (3 mg/L of CIP and 10 mg/L of GM) was similar to those referred to in the literature [31] to have high bactericidal activity (16 x MIC or 3 x MBC); however, it must be highlighted that a substantial number of viable cells remained adhered on PS surfaces. CIP is known for being initially very effective against *P. aeruginosa*. GM is also used to control *P. aeruginosa* growth and has been described in several works as a potential antibiotic to treat biofilm associated infections [32–34]. However, the diminished biofilm-forming capacity shown by PA and PAI under CIP and GM pressure may be related with other actions than bactericidal activity. Biofilm formation, by *P. aeruginosa* is hypothesized to follow a developmental pattern involving essentially four steps [35]: surface attachment, irreversible attachment, microcolony formation and differentiation into a mature population encased in a polymeric matrix. The presence of the antibiotics during biofilm formation may have interfered in the transition from reversible bindings to stable and irreversible interactions [36], affecting the transition from microcolonies to biofilms and thus delaying the mature biofilm development [37]. With the cessation of the antibiotic pressure, those less dense biofilms resumed their developmental process and gave rise to thicker biofilms, with again the PAI biofilms being superior to those formed by the reference strain.

During endoscopy procedure, the external environment surrounding the medical device provides optimal conditions for microbial adhesion and biofilm growth [6, 7]. If the disinfection procedures implemented during endoscopy reprocessing are not fully effective, biofilms may form and persist. These biofilms can later release bacterial cells that can spread to other locations, contaminating new surfaces and infecting the patients that underwent endoscopy. A vicious circle of biofilm growth, antimicrobial treatment, partial killing or inhibition of some susceptible population and regrowth of resilient cells can thus be created. This vicious circle was clearly observed in Figure 2, when biofilms obtained after the first period of PAE were submitted to a new cycle of antibiotic pressure with, a decrease in their density and activity being observed again. The number of biofilm-entrapped cells has also decreased but a substantial number of cells remained viable and adhered, mainly when GM was used. This latter event may be related with GM difficulties to diffuse across the biofilm matrix [38], limiting thus its access to the biofilm-growing cells. To augment its efficacy it has been recommended in-use concentrations of GM higher than those used to other antibiotics. Conversely, ciprofloxacin is known for its ability to penetrate rapidly [39]. Based on these data, it can be concluded that both antibiotics have great ability to disrupt established biofilms but poor capacity to completely inhibit biofilm-growing bacteria. Furthermore, when the level of antibiotics dropped, in the PAE periods, the population of adhered cells was able to multiply and to repopulate the biofilm observed during the second recovery period when the remaining biofilms recovered its metabolism becoming thicker.

This cycle of biofilm decrease/biofilm recovery may be explained by the existence, within the entire *P. aeruginosa* biofilm population, of a subpopulation of dormant cells that survive antibiotic treatment ensuring population survival. These cells that do not grow in the presence of an antibiotic, but neither do they die, are known as persister cells [40]. This subpopulation is recognized as “drug tolerant” as it remain metabolically inactive in stressful conditions, but it can resort to normal growth rates and susceptibility in the absence of antibiotic. The data gathered in this study allows speculating that biofilm development in the presence of antibiotics can be problematic as antimicrobial pressure can select persister cells and encourage bacterial adhesion and biofilm development. This resilient sessile population, that is normally able to sustain an antimicrobial attack, could account for the prevalence of biofilm-associated infections and for recalcitrance of surface contamination [40, 41].

The environmental pressure exerted by CIP and GM did not contribute to the development of *P. aeruginosa *tolerance to the same antibiotics. In fact, the 96-hour-old biofilms developed only in TSB are practically tolerant to the toxic action of CIP and GM; however, those biofilms formed under antibiotic selective pressure are to a certain extent susceptible to the same antibiotic attack. The sensitivity to the antibiotics of the biofilms formed by the isolated strain is similar to that displayed by the biofilms developed by reference strain. Based on this evidence, it can be referred that the environmental stresses to which the isolated *P. aeruginosa* have been submitted during endoscope reprocessing did not cause the development of a resistant phenotype towards the antibiotics studied. It is accepted that antimicrobial selective pressure may result not only in selection of persister cells but also in the development of cross-resistance towards other antimicrobials. In this study, data showed that BC was quite effective against *P. aeruginosa* biofilms, despite not having caused complete sanitation. All *P. aeruginosa* 96-hour-old biofilms developed or not under antibiotic pressure were similarly susceptible to BC attack (Figure 3). These data highlighted that biofilm-growing bacteria subjected to CIP and GM pressure did not exhibit cross-resistance to benzalkonium chloride. The use of this cationic surfactant is not advised in endoscope washing procedures, but this product is still used in clinical practice for surface disinfection, antisepsis preservation, and cleaning [42].

The exposure of cells within the biofilm to antibiotics pressure did not further promoted antimicrobial resistance to any of the antimicrobials tested (Figure 3). However, it must also be referred that none of the conditions caused complete sanitation of the biofilms, with, in general, the action of the antimicrobials being more effective in bacteria inactivation than in biofilm removal. This fact is of upmost importance as cells may then detach from the remaining biofilms and disseminate infection elsewhere.

The antimicrobial treatment of bacterial biofilms may lead to eradication of most of the susceptible or metabolically active population but again the small fraction of persister cells or bacteria in the deeper biofilm layers that are just exposed to subinhibitory concentrations can survive and be able to reconstitute the biofilm after discontinuation of antimicrobial therapy [29]. 

Since biofilms do not develop or mature in stress conditions but rather maintain a remaining adhered population and are exceptionally complex to eradicate, they are considered recalcitrant [38]. The persister cells give rise to a new diverse biofilm community with high genetic variability. This “new” biofilm is not as persistent as the cells that where in its foundation, being on the contrary as sensitive to external aggressions as a biofilm developed in normal conditions [38]. The persister cell role in biofilm survival will undoubtedly drive the effort to understand the mechanisms of their remarkable recalcitrance [43].

In this study, it was showed that the isolated endoscope strain possesses the ability to adhere in higher extent than the reference strain, developing after thicker biofilms. Its increased ability to adhere may be due to its previous stress exposure to cleaning agents and disinfection procedures. Moreover, biofilm development in the presence of high doses of antibiotics might lead to the eradication of the most part of the biofilm population, selecting just a small fraction of persister cells, which can survive, being able to reconstitute the biofilms following discontinuation of antibiotic therapy. This may represent an increased risk of infection to patients, requiring careful surveillance. As persister cells, which survive within the biofilm after treatments, can develop new biofilms and recolonize other accessible sites, the antibiofilm efficacy of a cleaning agent or antibiotic should not be just related with the reduction of biofilm mass or number of cells, but depends largely of its ability to kill all biofillm-cells, and also to completely eradicate biofilms from surfaces. 

## Figures and Tables

**Table 1:** Initial deposition rate (*j*_0_), number of adhered cells, (*n*_30 min _), number of adhered cells after PBS passage (*n*_60 min _), and percentage of detachment determined through the parallel plate flow chamber. Values are means ± SD for three measurements.

	*j*_0_ (10^3^ cells/(cm^2^ s))	*n*_30 min _ (10^7^ cells/cm^2^)	*n*_60 min _ (10^7^ cells/cm^2^)	Detachment (%)
*P. aeruginosa* ATCC 10145	3.9 ± 1.2	4.56 ± 0.8	3.62 ± 0.5	20.6
*P. aeruginosa* isolated strain	6.3 ± 2.0	7.49 ± 1.4	7.34 ± 1.2	2.0
